# Hypermethylation of the 5′ CpG island of the gene encoding the serine protease Testisin promotes its loss in testicular tumorigenesis

**DOI:** 10.1038/sj.bjc.6602373

**Published:** 2005-02-01

**Authors:** K J Manton, M L Douglas, S Netzel-Arnett, D R Fitzpatrick, D L Nicol, A W Boyd, J A Clements, T M Antalis

**Affiliations:** 1 Leukaemia Foundation and Cellular Oncology Laboratories, Queensland Institute of Medical Research, Queensland, Australia; 2 School of Life Science, Queensland University of Technology, Queensland, Australia; 3 School of Medicine, Southern Clinical Division, University of Queensland, Princess Alexandra Hospital, Queensland, Australia; 4 Department of Physiology, University of Maryland School of Medicine, Baltimore, MD, USA; 5 Amgen Inc., Seattle, WA, USA

**Keywords:** Testisin, methylation, serine protease, tumour suppressor

## Abstract

The Testisin gene (PRSS21) encodes a glycosylphosphatidylinositol (GPI)-linked serine protease that exhibits testis tissue-specific expression. Loss of Testisin has been implicated in testicular tumorigenesis, but its role in testis biology and tumorigenesis is not known. Here we have investigated the role of CpG methylation in Testisin gene inactivation and tested the hypothesis that Testisin may act as a tumour suppressor for testicular tumorigenesis. Using sequence analysis of bisulphite-treated genomic DNA, we find a strong relationship between hypermethylation of a 385 bp 5′ CpG rich island of the Testisin gene, and silencing of the Testisin gene in a range of human tumour cell lines and in 100% (eight/eight) of testicular germ cell tumours. We show that treatment of Testisin-negative cell lines with demethylating agents and/or a histone deacetylase inhibitor results in reactivation of Testisin gene expression, implicating hypermethylation in Testisin gene silencing. Stable expression of Testisin in the Testisin-negative Tera-2 testicular cancer line suppressed tumorigenicity as revealed by inhibition of both anchorage-dependent cell growth and tumour formation in an SCID mouse model of testicular tumorigenesis. Together, these data show that loss of Testisin is caused, at least in part, by DNA hypermethylation and histone deacetylation, and suggest a tumour suppressor role for Testisin in testicular tumorigenesis.

Testicular cancer is the most common malignancy affecting males aged 14–40 (Liu *et al*, 2000; Jemal *et al*, 2002) with the incidence of testicular germ cell tumours (TGCT) rising dramatically in recent years (Zheng *et al*, 1996). If diagnosed early, testicular cancer has a very high cure rate (Oliver, 1990, 2003). Nonetheless, there is a significant minority of TGCT that are resistant or become treatment resistant, suggesting that a better understanding of the molecular biology of this cancer would be valuable with regard to determinants of chemotherapeutic efficacy (Jones and Vasey, 2003).

TGCT commonly consist of two distinct histological subtypes, seminomas and nonseminomatous TGCT, with the latter including yolk sac, teratoma and mixed germ cell tumours. Somatic mutations leading to TGCT include chromosome duplication, loss of heterozygosity (LOH) and gene deletion (Sandberg *et al*, 1996; Lutzker and Barnard, 1998; Looijenga and Oosterhuis, 1999; Skotheim and Lothe, 2003). Epigenetic mechanisms are also increasingly recognised as a major mechanism of gene inactivation during TGCT progression. CG dinucleotide-rich regions, also known as CpG islands, in or near the proximal promoter regions of genes are targets for DNA methylation, leading to histone deacetylation, chromatin condensation and effective transcriptional silencing (Baylin *et al*, 1998; Herman, 1999). In normal cells, CpG methylation is an important mechanism for regulating gene expression, whereas in cancer cells, aberrant promoter methylation or hypermethylation can lead to abnormal gene silencing, including repression of tumour suppressor genes. An increasing number of tumour suppressor genes have been recognised for which epigenetic silencing is the predominant mechanism of gene inactivation and for which somatic mutations are rare. While several testicular tumour suppressor genes have now been identified (Bartkova *et al*, 2000; Datta *et al*, 2001; Luo *et al*, 2001; Eyzaguirre and Gatalica, 2002; Honorio *et al*, 2003), only a few, for example RASSF1A (Honorio *et al*, 2003), MGMT (Smith-Sorensen *et al*, 2002), have been reported to be silenced in testicular tumours through DNA hypermethylation.

Testisin was identified by virtue of its tissue-specific expression in testis and its absence in TGCTs, suggesting that Testisin may function as a tumour suppressor gene (Hooper *et al*, 1999). Chromosome 16p13, the region where the Testisin gene resides, is prone to mutations and deletions in multiple human cancers (Flint *et al*, 1997). The Testisin gene encodes a member of the serine protease family, a family of hydrolases that utilises the hydroxyl group of a serine amino-acid residue to cleave a target peptide bond (Rawlings and Barrett, 1994). Some members of this family cleave a wide range of target sequences, whereas others are quite narrow in their range of targets. Testisin belongs to a unique subgroup of trypsin-like serine proteases, which includes prostasin (Yu *et al*, 1994, 1995, 1996), murine and pancreasin (Bhagwandin *et al*, 2003). These enzymes are synthesised with a distinct carboxy-terminal peptide that is post-transcriptionally modified with a glycosylphosphatidylinositol (GPI)-membrane anchor (Inoue *et al*, 1998; Hooper *et al*, 1999; Honda *et al*, 2002; Netzel-Arnett *et al*, 2003). Thus, these enzymes may be present in plasma membranes or released from their anchor and secreted. The physiological function of Testisin is still not clear. In the case of prostasin, one role that has been identified is the regulation of epithelial Na+ channel (ENaC) function that is critical for normal salt and water balance (Vuagniaux *et al*, 2000; Tong *et al*, 2004).

The human Testisin gene (*TEST1; PRSS21*) is located within a cluster of serine protease genes on chromosome 16p13.3 (Inoue *et al*, 1999; Hooper *et al*, 2000) and consists of six exons ranging in size from 27 to 354 bp interspersed with five introns ranging in size from 97 to 1985 bp. Unusually for serine protease genes, the human Testisin gene contains a 5′ CpG island and a 5′ CpG rich region, which encompass the proximal promoter, transcription and translation initiation sites, the 5′ untranslated region and coding/noncoding sequences spanning exons I–III (Hooper *et al*, 2000). The Testisin 5′ proximal promoter lacks a TATA consensus sequence, but contains a putative CCAAT box, three consensus-binding sites for Sp1, two potential AP1 binding sites, one putative NF*κ*B binding site and several elements associated with testis-specific expression (Hooper *et al*, 2000). Many of these sites contain CpG dinucleotides located wholly within or bordering their sequence that may be affected by CpG methylation. In addition, the Testisin gene contains CpG dinucleotides located downstream of the transcription initiation site that, when methylated, may bind methyl-CpG binding proteins (MeCP), such as MeCP2 and MeCP1, which could contribute to transcriptional repression.

In the present study we have investigated the role of CpG methylation in Testisin gene inactivation and examined possible consequences for testicular tumorigenesis. Using the technique of sequence analysis of sodium bisulphite-treated genomic DNA (Clark *et al*, 1994), we find a strong correlation between hypermethylation of the Testisin 5′ region and loss of Testisin mRNA expression both in human tumour cell lines and in primary testicular tumour tissues. Additionally, inhibitors of gene methylation restore Testisin gene expression in Testisin-negative tumour lines. Finally, ectopic expression of Testisin in Tera-2 testicular tumour cells significantly suppressed tumorigenicity, providing the first functional evidence for Testisin as a suppressor of testicular tumour growth.

## MATERIALS AND METHODS

### Human tumour specimens and cell lines

Tissue from both normal and tumour-affected regions of orchidectomised testes were collected at the time of surgery, snap frozen in liquid nitrogen and stored at −80°C for subsequent RNA and genomic DNA isolation and analysis. Approval was obtained from the relevant institutional ethics committees. The Tera-2 teratocarcinoma cell line (NTera-2 clone 13) (Fogh *et al*, 1977; Thompson *et al*, 1984) and the embryonal carcinoma cell line GCT 27 C-4 were cultured in a mixture of MEM-alpha medium:HAMS F12 (1 : 1) (Invitrogen Australia, Pty Ltd, Victoria, Australia) supplemented with 10% foetal bovine serum (CSL, Parkville, Australia), 50 *μ*g ml^−1^ penicillin, 50 *μ*g ml^−1^ streptomycin, 25 mM HEPES and 15 *μ*g ml^−1^ 0.833% sodium hydroxide. The cervical carcinoma cell lines HeLa S3 (ATCC No. CCL-2.2) and SiHa (ATCC No. HTB-35) and the colon carcinoma cell line SW620 (ATCC No. CCL-227) were cultured in RPMI 1640 (Invitrogen Australia, Pty Ltd) supplemented with 10% foetal bovine serum, 50 *μ*g ml^−1^ penicillin and 50 *μ*g ml^−1^ streptomycin. All cell lines were cultured in 5% CO_2_ and 95% humidified air atmosphere at 37°C. Cell viability was determined by Trypan blue dye exclusion and mycoplasma-free status tested by Hoechst 33258 staining (Chen, 1977).

### RT–PCR and real-time PCR analyses

Total RNA was extracted using TRIZOL® reagent (Invitrogen, Victoria, Australia) as per the manufacturer's instructions. The RNA (2 *μ*g) was reverse transcribed using Superscript™ II reverse transcriptase (Invitrogen Australia, Pty Ltd). PCR was performed using 1–3 *μ*l of cDNA in a 25 *μ*l reaction containing 1 *μ*l (25 ng) of Testisin forward (5′-CTGACTTCCATGCCATCCTT-3′) and reverse (5′-GCTCACGACTCCAATCTGAT-3′) primer, 1 *μ*l (12.5 ng) of *β*-actin forward (5′-GACATGGAGAAGATCTGGCA-3′) and reverse (5′-ggtctttacggatgtcaacg-3′) primer, and 0.5 U of Red Hot DNA polymerase (Advanced Biotechnologies, Epsom, UK). The PCR program was as follows: 94°C for 5 min followed by 30 cycles of 94°C for 30 s, 56°C for 30 s and 72°C for 90 s with a final extension of 72°C for 10 min. PCR products were analysed by agarose gel analysis, purified using the Qiaex II gel extraction kit (Qiagen, Clifton Hill, Australia) and sequenced using the Testisin reverse primer and ABI PRISM BigDye™ Terminator Cycle Sequencing Reagent (Applied Biosystems, Foster City, CA, USA). PCR amplification of Testisin mRNA (460 bp product) was unaffected by the amplification of *β*-actin mRNA (375 bp product) in the same reaction mixture (data not shown).

For quantitative real-time PCR, specific primers and fluorescent-labelled probes for Testisin (Hs 00199035_m1) and *β*-actin (Hs 99999903_m1) were obtained from Assay-on-Demand Gene Expression Products (Applied Biosystems). cDNA was synthesised from RNA using the Taqman Reverse Transcription Reagents with random primers (Applied Biosystems) according to ABI optimised protocols (25°C for 10 min, 48°C for 30 min and 95°C for 5 min). PCR was performed using the ABI PRISM 7900HT sequence detector system. The thermal cycling conditions comprised an initial denaturation step at 95°C for 10 min followed by 40 cycles at 95°C for 15 s, and then 60°C for 1 min. Expression levels of *β*-actin were determined as an internal RNA control. Threshold cycle (*C*_T_) was obtained from PCR reaction curves. Three replicates each sample were analysed on two separate occasions to verify the precision of the assay. The relative quantification of Testisin was calculated using the comparative *C*_T_ method as recommended by Applied Biosystems with *β*-actin serving as the endogenous reference: Δ*C*_T_ (Testisin *C*_T_–*β*-actin *C*_T_); ΔΔ*C*_T_ (Δ*C*_T_−Δ*C*_T_ of untreated or control cell line), fold difference in target relative to untreated (2^−(ΔΔ*C*T)^).

### Sodium-bisulphite genomic DNA modification and sequence analysis

Genomic DNA from human tumour cell lines was extracted (Ciulla *et al*, 1988) and bisulphite modified as previously described (McDonald and Kay, 1997; Fitzpatrick *et al*, 1998). Genomic DNA from primary tissue specimens was bisulphite modified utilising low melting point agarose into which the DNA was embedded (Olek *et al*, 1996). The desulphonated bisulphite modified genomic DNA was amplified by two rounds of PCR using double nested oligonucleotide primers in a 59–50°C touchdown PCR protocol. The primers were designed to amplify a 385-bp region covering a CpG rich region (−245 to +115) overlapping the 5′ end of the Testisin CpG island (+25 to +873) (delineated using the Cpgplot algorithm which utilises the standard criteria of Gardiner-Garden and Frommer (Gardiner-Garden and Frommer, 1987)). The amplified region spans the proximal promoter, the 5′ untranslated region, the transcription initiation site and exon I (Hooper *et al*, 1999, 2000). The primers for the first round of PCR were forward 5′-TAGTTTGGGTAGAGATTTGGGGAGATTTTT-3′ (224F), reverse 5′-CTCCTACGACTCTACGAAAAACAAAAAATA-3′ (T845). The primers for the second round of PCR were forward 5′-GAAGGTTTTATGAAGGAGTAGTTATGTTTT-3′ (293F), reverse 5′-CTTCCTAAATCCAAGCCGAACCAACAACAA-3′ (733R). The samples were sequenced using the 733R oligonucleotide primer and ABI PRISM BigDye™ Terminator Cycle Sequencing Reagent (PE Applied Biosystems). All DNA samples were bisulphite modified and sequenced in two to five independent experiments.

A number of controls were included to monitor the efficiency of sodium bisulphite modification and to ensure a lack of bias in the PCR reactions. To verify that bisulphite treatment resulted in complete conversion of unmethylated cytosines to uracil in each genomic DNA sample, the complete conversion of every cytosine not contained within a CpG site to uracil regardless of the methylation status of the gene was monitored. Genomic DNA isolated from HeLa and SW620 cells were included as unmethylated and methylated controls respectively, as part of each experiment to monitor PCR bias in the selective amplification of unmethylated over methylated sequences or *vice versa* (Warnecke *et al*, 1997). Bisulphite conversion was also conducted on mixtures of methylated and unmethylated controls (i.e. 50% HeLa genomic DNA/50% SW620 genomic DNA) to monitor bias in semimethylated samples. These mixtures were sequenced to reproduce the expected semimethylated signal on multiple occasions (data not shown). The methylation status of the coding strand was determined for all samples; verification of the methylation status on the noncoding strand was performed for a subgroup of genomic DNA samples and found to be consistent in all cases (data not shown). The oligonucleotide primers used in the first round PCR amplifying the noncoding DNA strand were forward 5′-GCGATTTTGCGGGAGATAAGAAGTGATTTT-3′ (TC535), reverse (TC1160) 5′-CAATAAAAACAACCTAAACAAAAACTTAAA-3′. The primers for the second round PCR were forward 5′-TCGAGTTTATTCGGTTTTTTGAGTTTAGTT-3′ (TC628), reverse 5′-AAAACTTAAAAAAATCCCCATTCTAAATAA-3′ (TC1130).

### *In vitro* demethylation and histone deacetylase inhibition of human tumour cell lines

The human tumour cell lines Tera-2, SW620 and GCT27C-4 were seeded at low density (2.5 × 10^5^ cells), allowed to adhere overnight, then treated with either 2–10 *μ*M 5-azacytidine (5-aza), 10–100 nM trichostatin A (TSA), 1–10 *μ*M 5-aza-2′-deoxycytidine (5-aza-2′) or combinations of these reagents for 3–4 days (2 days for TSA) with the media changed every 24 h. RT–PCR analysis as described above using Testisin and *β*-actin oligonucleotide primers was performed on cellular RNA.

### Generation of stable transfectants

Full-length Testisin cDNA in pcDNA3 (Hooper *et al*, 1999) was transfected into Tera-2 cells by electroporation (250 mV, 960 *μ*F). Clones resistant to G418 (0.1 mg ml^−1^) (Invitrogen Australia, Pty Ltd) were isolated and screened for Testisin mRNA expression by RT–PCR as described above. Four independent clones expressing Testisin mRNA (C1, C11, C29 and C33) and a cell line containing the pcDNA3 vector alone (VOC2) were selected for subsequent experiments.

### *In vivo* orthotopic testicular tumour model

This model was performed essentially as we have published previously (Douglas *et al*, 2001) using 7–8-week-old severe combined immune deficient (SCID) mice (6–8 mice per group; C.B.-17-SCID) (Australian Resource Centre, Perth, Western Australia, Australia). Mice were housed in a temperature-controlled, specific pathogen-free room (23°C, 12 h light–dark regime) with free access to water and a standard diet (Norco, Lismore, NSW, Australia). Tumour cells were implanted when the mice were 8 weeks of age. The study was approved by the Group 5 Animal Experimental Ethics Committee of the University of Queensland and performed according to NHMRC Guidelines (AEEC Approval Number SURG/394/98, SURG/569/99; BCREC Approval A9806-01-019M). Under general anaesthesia following exteriorisation of the testes, Tera-2 clones isolated above were injected into the left testis (1 × 10^6^ cells in 25 *μ*l PBS). As an internal control, the contralateral (right) testis was either injected with an equal volume of PBS or not injected; no difference was detected between the two methods. After 4 weeks, or when the apparent tumour size approached 1 cm^3^, both testes were removed, weighed and half was fixed in formalin for histological analyses and the remaining half, frozen in liquid nitrogen for RT–PCR analyses. The lungs were inflated with Bouin's fixative and removed for histological analyses. Testes weights were represented as a percentage of total body weight in grams to correct for differences in size between individual mice. Tumour burden was calculated by dividing the value for the tumour-affected left testis by that of the contralateral testis. These studies were performed according to the Australian ‘Code of Practice for the Care and Use of Animals for Scientific Purposes’. During the 4-week period of tumour development, the mice exhibited no symptoms or signs of clinical illness apart from a visible or palpable lump in the lower abdomen or scrotum.

### Immunohistochemistry

Paraffin sections (3–4 *μ*m) of mouse tissues were affixed to adhesive slides and air-dried overnight at 37°C. For immunohistochemistry, sections were deparaffinised and immersed in methanol containing 0.3% H_2_O_2_ for 30 min to exhaust endogenous peroxidase activity. After thorough washing, the sections were preincubated with 10% horse serum, followed by anti-Testisin monoclonal antibody (DD-P104 C37; diaDexus, Inc., South San Francisco) at 8 *μ*g ml^−1^ for 1 h at room temperature. After washing in PBS, biotinylated anti-mouse IgG was applied for 30 min at room temperature. The sections were washed thoroughly in PBS before incubating in Vectastain ABC reagent for 30 min at room temperature. Sections were developed by incubation in 0.05% 3,3′-diaminobenzidine (DAB) in Tris-HCl, pH 7.4 buffer with H_2_O_2_ as substrate. After washing in water, the sections were lightly counterstained with Mayer's haematoxylin. Negative controls were stained as above but with PBS substituted for the primary antibody. Histology sections were stained with Mayer's haematoxylin and eosin.

### *In vitro* cell proliferation assay

Tera-2 cells were seeded in 96-well tissue culture plates (Costar) in triplicate at low (1000 cells), medium (5000 cells) and high (10 000 cells) density and allowed to grow for 2, 3 or 4 days under normal culture conditions. Cell proliferation was assayed by 5-bromo-2′-deoxyuridine (BrdU) (colorimetric) ELISA (Roche) as per the manufacturer's instructions. The assay was replicated on four separate occasions.

### *In vitro* colony forming assays

For monolayer assays, cells were plated in six-well plates (100 cells per well) in triplicate and cultured for 14 days with the media changed every 4 days. The cells were fixed, stained with 1% crystal violet and colonies of greater than 50 cells were counted. For assay of colony formation in soft agarose, cells were embedded in 0.33% agarose, which was sandwiched between a 0.6% agarose base and a 0.33% top layer with media, in triplicate in six-well plates. Plates were incubated for 4 weeks under normal culture conditions. The number and approximate colony sizes were recorded.

### Statistical analyses

The nonparametric Mann–Whitney test was used to determine differences between two groups, and the nonparametric Kruskal–Wallis test was used for the analysis of differences among more than two groups. *P*<0.05 was considered statistically significant.

## RESULTS

### Testisin gene silencing correlates with DNA hypermethylation in human tumour cell lines

**Figure 1:**
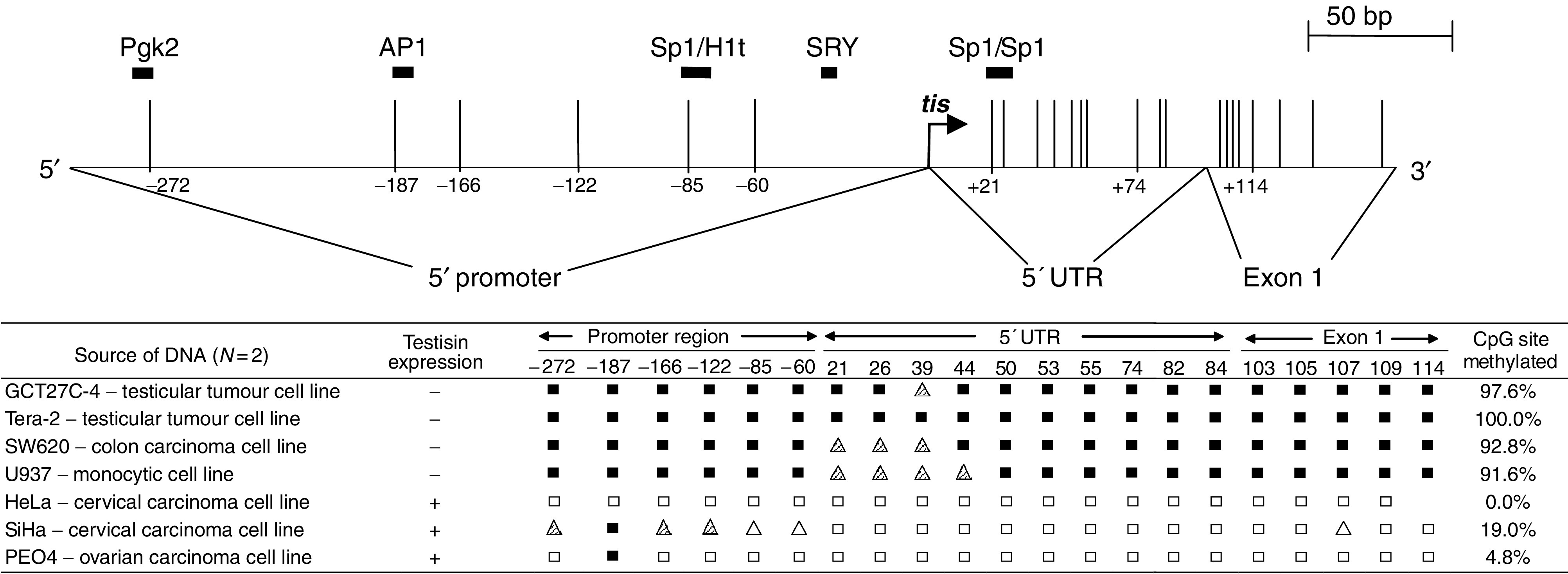
Testisin expression correlates with DNA hypermethylation in human tumour cell lines. The upper diagram shows a schematic illustration of the Testisin 5′ CpG island analysed by bisulphite sequencing. Thin vertical bars map the location of 21 CpG sites analysed within this region. The transcription initiation site (*tis*) is indicated by the right-angled arrow. Sites that conform to the consensus binding elements for Sp1, AP1 and SRY, and the regulatory elements associated with transcription of testis-specific proteins H1t and Pgk2 are indicated by bold black boxes. The table shows the results of sequence analysis within the Testisin 5′ CpG island of bisulphite treated genomic DNA isolated from non-Testisin expressing tumour lines (GCT27C-4, Tera-2, SW620 and U937) and Testisin expressing tumour lines (HeLa, SiHa and PEO4). Expression of Testisin mRNA (indicated by +) was determined by RT–PCR (data not shown). The percentage of CpG sites methylated per cell line was calculated by adding the score of each CpG site and dividing by (the total number of sites analysed multiplied by 2), giving a maximum total of 42. The scoring and representative symbols are as follows: fully methylated CpG site (black shaded square; score=2); fully unmethylated CpG site (unshaded square; score=0); a semimethylated CpG site where the methylated signal is stronger than the unmethylated signal (hashed triangle; score=1.5); a semimethylated CpG site where the unmethylated signal is stronger than the methylated signal (unhashed triangle; score=0.5). The data represents a 3–5 independent bisulphite sequencing experiments.

As reported previously (Hooper *et al*, 1999), the Testisin gene contains a 5′ CpG island and an overlapping CpG rich region spanning nucleotides −245 to +873 which encompasses the Testisin proximal promoter, the 5′-untranslated region and the beginning of exon I (Figure 1, top). To investigate whether Testisin expression may be associated with the methylation status of the 21 CpG dinucleotides in the 385 bp 5′ CpG island, sodium bisulphite sequence analysis was utilised. This technique enables the methylation status of individual CpG sites in a region to be determined by selective conversion of unmethylated cytosines, but not methylated cytosines, to uracil, following treatment of the DNA with sodium bisulphite. Analysis of genomic DNAs isolated from Testisin-expressing (HeLa, SiHa and PEO4) and non-Testisin expressing (GCT27C-4, Tera-2, SW620 and U937) tumour cell lines showed that Testisin expressing cell lines contained very few methylated CpG sites (<20%) within this region, whereas the non-Testisin expressing cell lines showed a high percentage (>90%) of fully methylated CpG sites (Figure 1). These data show that Testisin gene expression correlates with hypermethylation of the CpG sites within this 385 bp region and point to a role for CpG methylation in the regulation of Testisin gene expression.

### The Testisin gene is hypermethylated in primary TGCT

**Figure 2:**
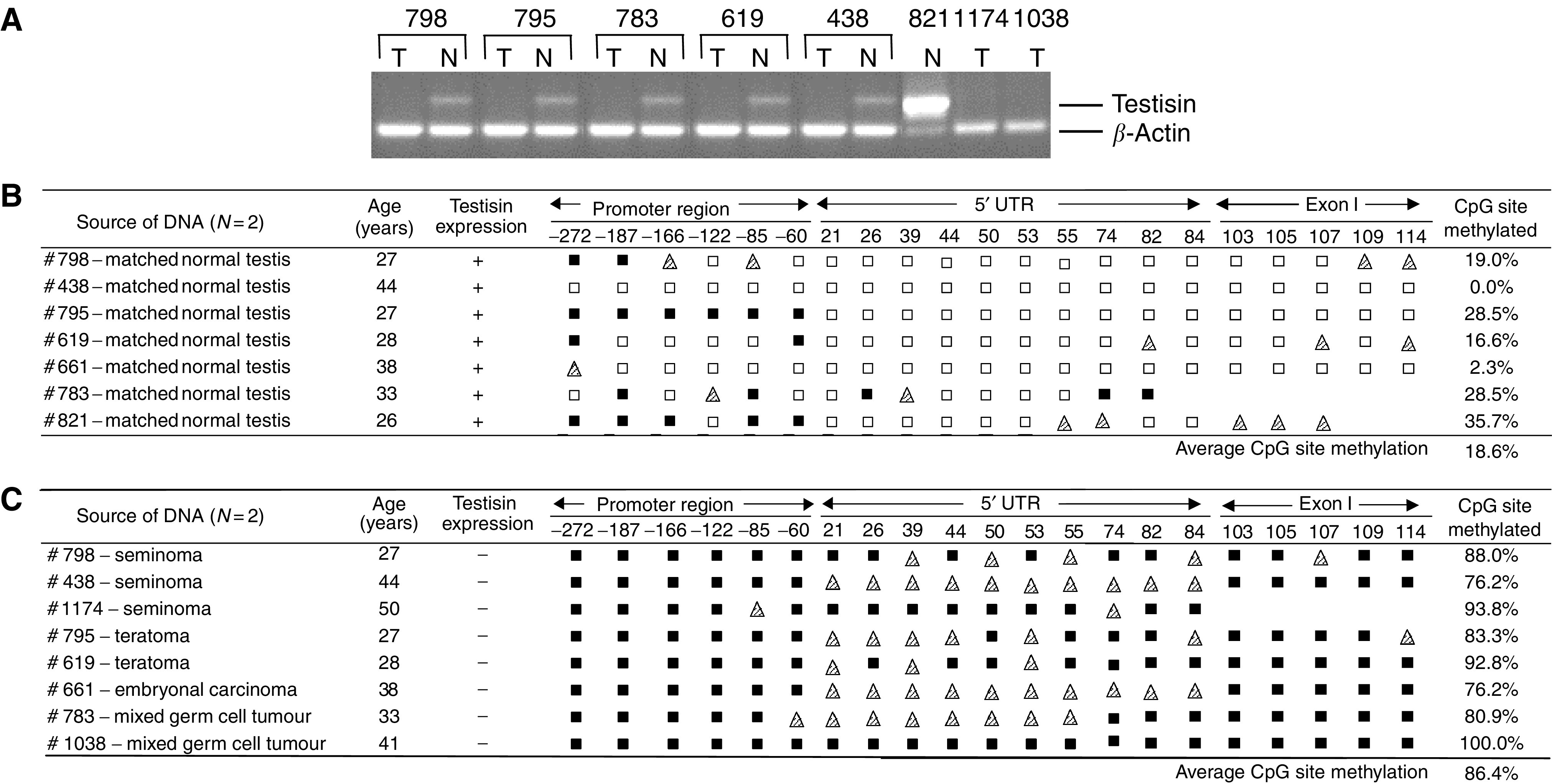
Silencing of the Testisin gene in testicular germ cell tumours is associated with DNA hypermethylation. (**A**) Testisin mRNA expression in representative primary human tissue specimens. RNA isolated from testicular tumour (T) and unaffected adjacent normal tissues (N) from individual patients was evaluated by RT–PCR for Testisin mRNA expression. *β*-Actin mRNA was monitored as a measure of mRNA concentration and integrity. The tables show the results of sequence analysis of the Testisin 5′ CpG island of bisulphite treated genomic DNA isolated from matched primary tissues from individual patients: unaffected ‘normal’ testicular tissue adjacent to tumour (**B**) and testicular tumour tissues (**C**). The symbols and the percentage methylated CpG sites were calculated as described in the legend to Figure 1. The data are representative of a minimum of two independent bisulphite sequencing experiments.

We have demonstrated previously that while Testisin is present in testicular spermatocytes, TGCTs lack Testisin expression (Hooper *et al*, 1999). Detection of Testisin mRNA by RT–PCR amplification of RNA isolated from seven sets of primary tissue specimens (consisting of tumour and adjacent normal tissues from the same patient) confirmed the absence of Testisin mRNA in the testicular tumour tissues (Figure 2A). To determine whether the silencing of Testisin gene expression in the testicular tumours was associated with methylation of the 385 bp 5′ CpG region, sodium bisulphite sequencing analysis was performed on genomic DNA isolated from these tissue specimens. The majority of the CpG sites within the Testisin 5′ CpG island in the normal, unaffected testis tissues were unmethylated (an average of 18.6% methylated CpG sites) (Figure 2B). The 5′ untranslated region and exon I contained predominantly unmethylated CpG sites, with only three specimens showing evidence of partial methylation. The proximal promoter region was largely unmethylated but similarly contained some methylated CpG sites. Unusually, genomic DNA from one patient (#795) showed methylation of all six CpG sites within this region, despite evidence of Testisin mRNA expression by RT–PCR. In the testicular tumour tissues, the majority of the CpG sites were methylated (an average of 86.4% of methylated CpG sites) (Figure 2C). There appeared to be an increased number of semimethylated CpG sites in the 5′ untranslated region of the Testisin gene in the tumour specimens compared with the large number of methylated sites in the proximal promoter and exon I. Thus, the Testisin gene methylation patterns and silencing observed in testicular tumour cell lines reflects aberrations present in primary testicular tissues. Taken together, these data demonstrated a clear inverse correlation between the methylation status of the Testisin 5′ CpG region and expression of the Testisin gene in both tumour cell lines and primary testicular tumours. These results suggest that hypermethylation of the Testisin gene is involved in silencing of the Testisin gene in TGCT.

### Reactivation of Testisin mRNA expression by demethylation and histone deacetylase inhibition

**Figure 3:**
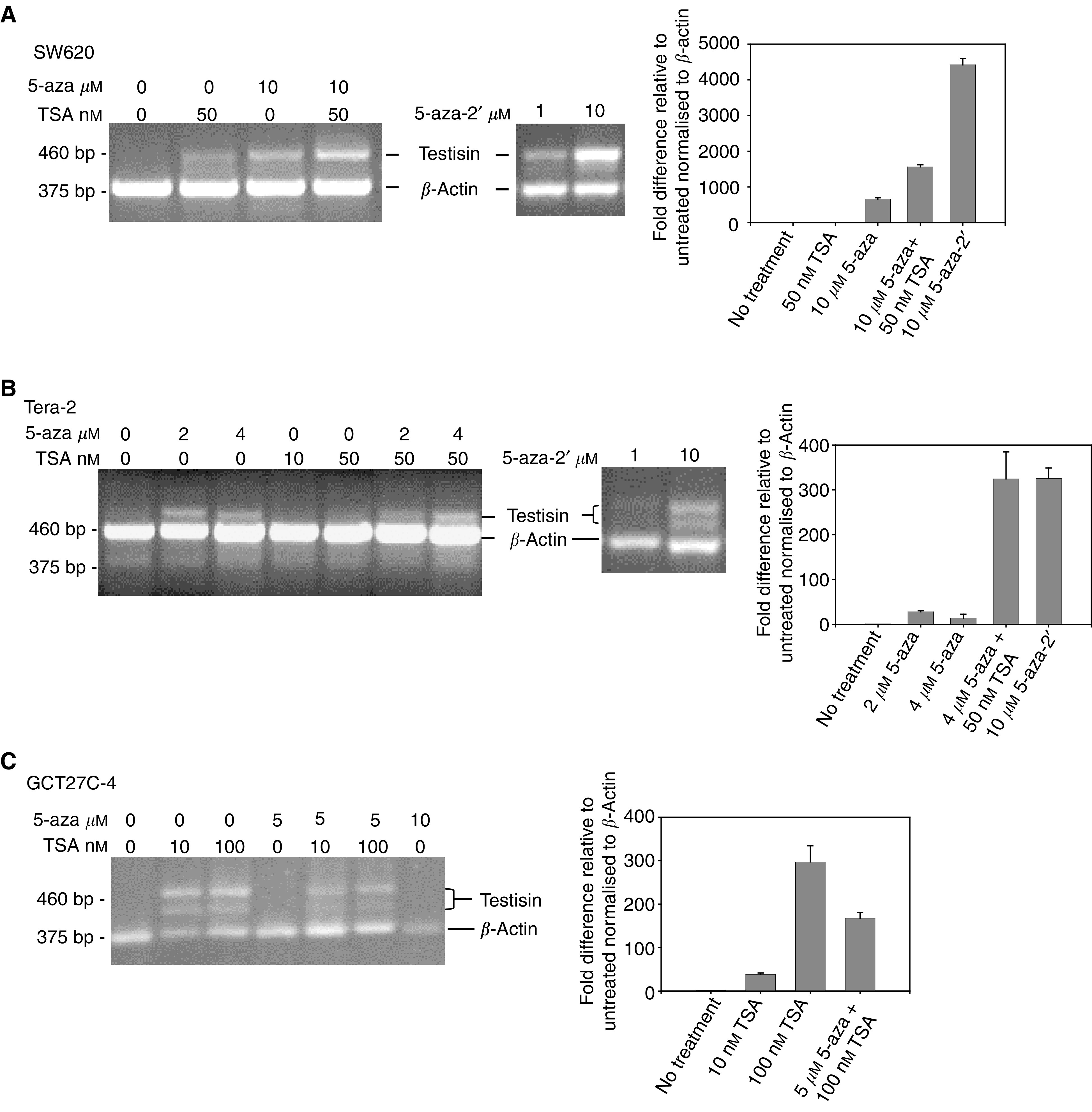
Reactivation of the Testisin gene following demethylation and histone deacetylase inhibition. Reactivation of Testisin in SW620 cells (**A**), Tera-2 cells (**B**), and GCT27C-4 cells (**C**) after treatment with 2–10 *μ*M 5-azacytidine (5-aza), 1–10 *μ*M 5-aza-2′-deoxycytidine (5-aza-2′) and/or 10–100 nM Trichostatin A (TSA) alone or in combination. RT–PCR was performed using 3 *μ*l of cDNA in the presence of Testisin and *β*-actin oligonucleotide primers for 40 cycles at 56°C annealing temperature. Two PCR products encoding Testisin are detected in Tera-2 and GCT27C-4 cells due to the expression of an alternate splice variant, which is missing the terminal 40 bp of exon 5 (determined by DNA sequence analysis, data not shown). Representative results from 2–4 independent experiments are shown. To the right of the DNA gels are shown the results of quantitative analysis of Testisin mRNA transcripts under each given condition as determined by real-time quantitative PCR. A value of 1 is arbitrarily set for the untreated control cell line. The data is representative of two independent experiments performed in triplicate.

Methylated promoter DNA may interfere with binding of transcription factors or other CpG binding proteins to silence gene transcription, and histone deacetylases may act synergistically in conjunction with DNA methylation to co-repress gene expression (Cameron *et al*, 1999). It has been found that expression of genes silenced by promoter methylation may be restored by treatment of cells with the demethylating agents 5-aza or 5-aza-2′-deoxycytidine (5-aza-2′), and combined treatment with a histone deacetylase inhibitor can further stimulate re-expression (Esteller, 2002). To investigate whether the Testisin gene may be reactivated following demethylation of CpG sites and/or histone deacetylase inhibition, Testisin-nonexpressing tumour cell lines were treated with the demethylating agents, 5-azacytidine, 5-aza-2′-deoxycytidine, and/or the histone deacetylase inhibitor TSA. Testisin mRNA expression was followed by RT–PCR analysis and co-amplification of *β*-actin mRNA confirmed the quality of the total RNA used in each reaction. The upregulation of Testisin mRNA expression following treatment was quantitated by real-time RT–PCR. Testisin mRNA was induced in SW620 cells after treatment with 10 *μ*M 5-azacytidine (Figure 3A), and was induced in Tera-2 cells following treatment with as low as 2 *μ*M 5-azacytidine (Figure 3B). The demethylating agent, 5-aza-2′-deoxycytidine, also induced Testisin mRNA expression in both SW620 (Figure 3A) and Tera-2 cells (Figure 3B) at 10 *μ*M. TSA induced very low to negligible levels of Testisin mRNA expression in SW620 cells, which increased when TSA was used in combination with 5-azacytidine (Figure 3A). TSA alone did not induce Testisin mRNA in Tera-2 cells, but was more effective in combination with 5-azacytidine (Figure 3B). GCT27C-4 cells were sensitive to TSA treatment, with Testisin mRNA expression observed after treatment with 10 and 100 nM TSA (Figure 3C). Interestingly, 5-azacytidine appeared to have a negative effect on the upregulation of Testisin mRNA induced by TSA in these cells. The presence of doublet Testisin bands in Tera-2 and GCT27C-4 cells is due to a splice variant of the Testisin transcript, resulting in the deletion of 40 bp of exon 5 (data not shown). This Testisin isoform has been reported previously (Inoue *et al*, 1999), although its significance is not known. In all cases, Testisin mRNA re-expression was accompanied by partial demethylation of the Testisin 5′ CpG region as determined by bisulphite sequencing (data not shown). These data further confirm that Testisin is silenced in testicular tumour cells by hypermethylation.

### Expression of Testisin mRNA reduced the tumorigenicity of Tera-2 cells in an orthotopic xenograft model of testicular tumorigenesis

Our findings show that Testisin mRNA expression is silenced in testicular tumour cells through a mechanism involving DNA hypermethylation of the Testisin gene. To investigate whether inactivation of the Testisin gene may confer a selective advantage to testicular tumorigenicity, the effect of expression of the Testisin gene in Tera-2 cells, which lack Testisin gene expression and form tumours when implanted in an orthotopic model of testicular tumorigenesis (Douglas *et al*, 2001), was investigated.

**Figure 4:**
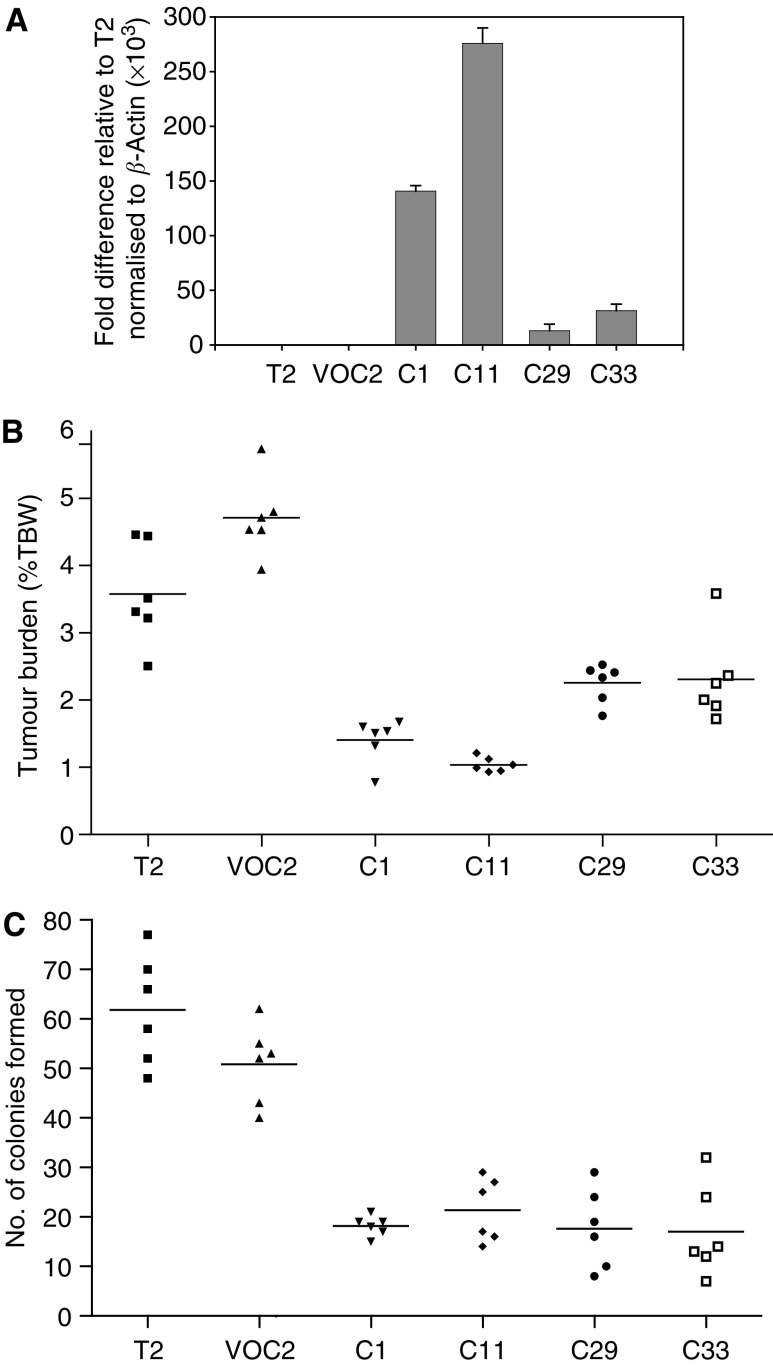
Expression of the Testisin gene suppresses tumorigenicity of Tera-2 cells *in vivo* and inhibits anchorage-dependent colony formation *in vitro*. (**A**) Testisin mRNA expression in transfected Tera-2 cell lines. Graph showing fold difference in Testisin mRNA expression levels relative to *β*-actin determined by quantitative real-time PCR: mRNA isolated from parental Tera-2 cells [T2], Tera-2 clones transfected with Testisin cDNA [C1, C11, C29, C33] or the control Tera-2 clone transfected with vector alone [VOC2]. (**B**) Testisin gene expression suppresses Tera-2 tumour growth in a murine *in vivo* orthotopic xenograft model of testicular tumorigenesis. Tumour burden (testis tumour weight as a percentage of the total mouse body weight) was calculated as described in the Materials and Methods and is displayed as a scattergram with the line centered on the mean of the values. C1, C11, C29, C33 *vs* VOC2, *P*=0.0022; Kruskal–Wallis nonparametric test. There was no significant difference between parental Tera-2 cells and the vector alone control, T2 *vs* VOC2, *P*=0.0877. (**C**) Testisin gene expression inhibits anchorage-dependent colony forming ability of Tera-2 cells *in-vitro*. Cells were plated at low density and colony formation monitored over 14 days with media changed every 4 days. C1, C11, C29, C33 *vs* VOC2, *P*=0.0022; Kruskal–Wallis nonparametric test. There was no significant difference between parental Tera-2 cells and the vector alone control, T2 *vs* VOC2, *P*=0.1320.

An expression plasmid containing Testisin cDNA or the control vector alone were transfected into Tera-2 cells and stable cell lines isolated on the basis of G418 resistance. Four independent clones, C1, C11, C29 and C33, expressing Testisin mRNA (Figure 4A) and a vector alone control (VOC2) were selected and used for tumorigenicity studies. Clones expressing Testisin mRNA suppressed the tumorigenicity compared with the Tera-2 parent line and the vector alone control Tera-2 cells (Figure 4B). The observed growth suppression appeared to correlate with Testisin mRNA expression level; clones C1 and C11 showed the highest Testisin mRNA expression and the least tumour burden. Growth suppression was replicated in three independent experiments. After the 4-week period of *in vivo* tumour growth, all tumours were removed and Testisin expressing clones shown to have maintained human Testisin mRNA expression by RT–PCR (data not shown).

**Figure 5:**
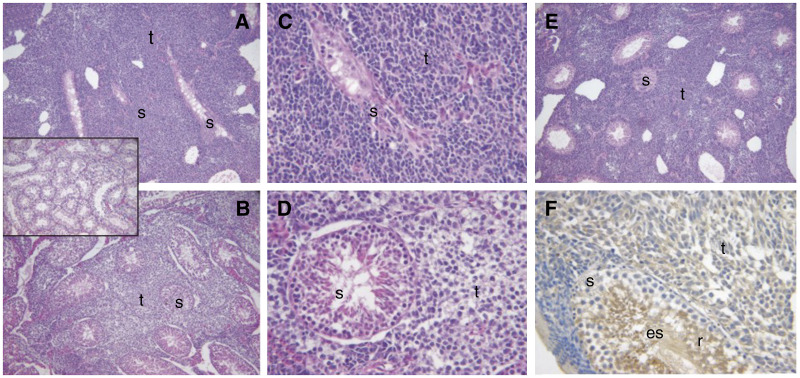
Photomicrographs of histological analyses of murine orthotopic testicular tumours. Tissue sections were stained with Mayers’ haematoxylin and eosin. (**A**, **B**) Examples of severely atrophied residual seminiferous tubules (s) present in testes containing testicular tumours (t) formed after 4 weeks following injection of the vector alone cell line control. (**A**) and (**B**) are × 100 and × 400 magnification respectively. (**C**) Representative residual seminiferous tubule with moderate atrophic changes present in testes 4 weeks following injection of C33 (× 100 magnification). (**D**, **E**) Examples of residual seminiferous tubules with mild atrophic changes after 4 weeks following injection of C11, at magnifications of × 100 and × 400, respectively. (**F**) Immunohistochemical staining for Testisin in a section containing C11 tumour at × 400 magnification. The monoclonal antibody (DD-P104 C37) reacts with human Testisin present in the tumour (t) as well as murine Testisin present in the round (r) and elongating spermatids (es) of the murine seminiferous tubules. The inset (× 100 original magnification) shows a contralateral testis with normal morphology.

Morphological analysis of tissue specimens showed that growth of the parental Tera-2 cells or the control (vector alone) tumour cells resulted in virtual replacement of the affected testis with the growing mass of tumour cells. Tumour growth blocked the blood supply to the seminiferous tubules causing widespread severe atrophic changes in residual seminiferous tubules (Figures 5A and B). Each of the Testisin mRNA expressing Tera-2 clones (C1, C11, C29 and C33) demonstrated reduced tumour burden relative to the parental and vector only lines, and visibly displaced less of the testis causing fewer disruptions to the blood supply. Moderate atrophy was seen in the seminiferous tubules surrounding several clones (for example, Figure 5C). Only mild atrophy was seen in the seminiferous tubules for C11 (Figures 5D and E). Stained sections of contralateral (right) testes showed normal morphology independent of tumour growth in the left testis (inset Figure 5). Testisin staining of tumour sections using an anti-Testisin monoclonal antibody showed light, patchy staining of tumour tissue relative to Testisin staining of germ cells (Figure 5F). No lung metastases were detected in any of the experimental animals, independent of Testisin expression in the testicular tumours (data not shown).

### Re-expression of Testisin mRNA inhibited *in vitro* colony formation

The suppression of Tera-2 tumour growth *in vivo* suggests that the Testisin gene may function as a tumour suppressor. Expression of Testisin mRNA did not affect Tera-2 *in vitro* cell proliferation as assessed by BrdU incorporation (data not shown). In addition, no alterations in cell viability or cell morphology under normal culture conditions *in vitro* were observed (data not shown). As *in vitro* growth of Tera-2 cells is anchorage-dependent, the effect of Testisin mRNA expression on Tera-2 malignant potential *in vitro* was examined by colony forming assay in monolayer. Tera-2 clones expressing Testisin mRNA formed fewer colonies than the parent line or pcDNA3 vector only clones (*P*<0.005) (Figure 4C), consistent with the suppressed tumorigenicity observed *in vivo*. Testisin mRNA expression in Tera-2 cells did not confer anchorage-independent growth as measured by colony formation in agarose (data not shown).

## DISCUSSION

The Testisin gene is specifically expressed by meiotic germ cells in human testis (Hooper *et al*, 1999), and is absent in TGCT (Hooper *et al*, 1999), suggesting it is a candidate tumour suppressor gene for testicular tumours. Here we demonstrate that the Testisin gene is regulated by DNA methylation and that hypermethylation of the Testisin 5′ CpG island represents a principal mechanism for inactivating this gene in testicular cancers (Figures 1 and 2). Further we provide the first functional evidence that re-activation of Testisin gene expression, through exogenous expression of Testisin cDNA, suppresses *in vivo* testicular tumour growth and colony forming ability *in vitro*, supporting the hypothesis that Testisin inactivation confers a selectable advantage for testicular tumours.

That the Testisin gene is silenced by DNA hypermethylation was demonstrated by (i) the correlation of CpG island methylation status with gene expression in tumour cell lines and in primary testicular tumour tissues (Figures 1 and 2) and (ii) re-expression of Testisin mRNA following demethylation and histone deacetylase inhibition (Figure 3). Thus, Testisin may be added to an emerging list of tumour suppressor genes that are silenced by DNA hypermethylation, including prostasin (Chen and Chai, 2002), E-cadherin (Melki *et al*, 2000; Nakayama *et al*, 2001), normal epithelial cell-specific 1 (NES1) (Li *et al*, 2001) and COX-2 (Kikuchi *et al*, 2002).

Mechanisms of silencing of gene transcription by DNA methylation occur either through methylation of specific CpG dinucleotides contained within the binding sites for transcription factors (Tate and Bird, 1993) or through methylation of a region of CpG dinucleotides resulting in binding of methyl CpG proteins to the DNA that in turn inhibit access of regulatory elements to the DNA and inhibit transcription (Boyes and Bird, 1992). The Testisin 5′ CpG island contains four CpG dinucleotides located wholly or partially within the binding sites for transcription factors Sp1 [(+20 to +26); (+26 to +31); (−86 to −81)] and AP1 (−184 to −190) and the testis-specific regulatory element, CCCCACCC (−279 to −272) that is homologous to that found in the Pgk2 gene (Hooper *et al*, 2000). Furthermore, the Sp1 site at (−81 to −86) is required for minimal Testisin promoter activity in HeLa cells as demonstrated by reporter gene assay (Inoue *et al*, 1999). This may indicate that the methylation status of these four CpG dinucleotides plays a primary role in Testisin gene regulation. However, the present studies suggest that while partial demethylation was detected in the proximal promoter region of the Testisin gene, there was consistently more demethylation of CpG dinucleotides within the 5′ untranslated region (Figures 1 and 2B). It has been proposed that methylation of CpG dinucleotides near transcription initiation sites may strongly inhibit gene transcription by inhibiting the setting of the basal transcription machinery (De Smet *et al*, 1999). This mechanism of gene suppression has been suggested for other genes, for example, *HPRT*, *PGK1,* which, like *PRSS21*, contain CpG-rich TATA-less promoters (De Smet *et al*, 1999). Thus, the Testisin 5′ untranslated region may play a primary role in silencing of gene transcription, possibly via binding of methyl CpG proteins to CpG dinucleotides in the vicinity of the transcription initiation site. The synergy between the demethylating agents and histone deactylase inhibitors to reactivate Testisin gene expression indicates that in addition to DNA methylation and the methyl CpG proteins, other indirect epigenetic mechanisms, such as histone modifying enzymes and chromatin, are working together to silence Testisin gene expression.

The semimethylated CpG sites observed in the 5′ untranslated region of testicular tumour tissues may reflect some heterogeneity in these tissues. It is likely the tissues are comprised of mixed tumour cell types and also infiltrating cells, such as inflammatory cells and lymphocytes that may contribute to the heterogeneity (Heidenreich *et al*, 1998). Alternatively, the semimethylated CpG sites may indicate that not all CpG sites within the Testisin 5′ untranslated region need to be fully methylated for the Testisin gene to be silenced. It was surprising that the Testisin 5′ CpG island was essentially unmethylated in normal testis, given that the Testisin gene is only expressed by a small population of cells, the meiotic spermatocytes among the germ cells at various stages of maturation (Hooper *et al*, 1999), and not by other somatic cells present in the testis (Sertoli and Leydig cells). It is probable that mechanisms other than DNA methylation are responsible for the cell- and maturation-specific regulation of the Testisin gene. Thus, epigenetic gene silencing, reflected by the observed hypermethylation of the Testisin 5′ CpG island, represents a mechanism that appears to be tumour-specific.

While hypermethylation of the Testisin 5′ CpG island is most likely responsible for the silencing of Testisin in testicular tumours, it is unclear whether this represents a cause or consequence of testicular tumorigenesis (Herman, 1999). Knudson's two hit theory of tumour-suppressor gene inactivation proposes inactivation of one allele by a mutation or epigenetic event followed by inactivation of the second allele by a large chromosome deletion or other event during tumour growth (Knudson Jr, 1986). Hypermethylation of tumour DNA has been shown to occur in chromosomal regions that undergo LOH, suggesting a causative link between the two phenomena (Baylin *et al*, 1991). Hypermethylation of the Testisin 5′ CpG island and the loss of Testisin expression in TGCT suggest that hypermethylation of the Testisin gene could be functionally relevant for testicular tumorigenesis.

The finding of gene silencing associated with hypermethylation has precedents with two other protease genes. The NES1 serine protease gene is downregulated in breast and prostate cancers, and its expression is associated with a tumour suppressor function (Goyal *et al*, 1998; Luo *et al*, 2001). The prostasin gene is downregulated in prostate cancers and its expression functionally inhibits both prostate (Chen *et al*, 2001, 2004) and breast cancer invasiveness (Chen and Chai, 2002). Similar to our observations on the Testisin gene, hypermethylation of both prostasin and NES1 genes has been reported as the basis for loss of expression of these genes in tumours (Li *et al*, 2001; Chen and Chai, 2002), and their effects on cells are not associated with reduced proliferation (Goyal *et al*, 1998; Chen *et al*, 2001). Taken together, these findings support an emerging new paradigm for serine protease genes wherein they may be silenced by epigenetic mechanisms leading to enhanced tumorigenesis. In contrast to the inhibitory effect of Testisin expression on testicular tumorigenesis, Testisin overexpression has been associated with advanced stage disease in ovarian carcinomas (Shigemasa *et al*, 2000). These data indicate that the effect of Testisin on tumour growth and survival are further dependent on the cellular context and tumour microenvironment. Cell surface expression of a protease by a tumour cell will lead to activities dependent on available proteolytic targets, and these may be quite different in different tissue environments, particularly if the protease is not normally expressed in that tissue.

The data presented here demonstrate that Testisin gene silencing is associated with hypermethylation of the Testisin CpG island in primary testicular cancers and support a role for Testisin as a tumour suppressor in testicular cancers. The inactivation of a tumour suppressor gene through epigenetic mechanisms leaves the gene structure intact and provides for the therapeutic possibility that the gene can be re-activated *in vivo* allowing for the ‘tumour suppressor’ function of the gene to be restored (reviewed in Esteller, 2002). This *in vivo* re-activation is currently being used in the clinical setting to re-express foetal haemoglobin to treat sickle cell anaemia (Koshy *et al*, 2000). The re-expression of Testisin and other tumour suppressor genes could be initiated by a similar treatment if issues relating to toxicity could be resolved (Christman, 2002).
